# Band Alignment and Controllable Electron Migration between Rutile and Anatase TiO_2_

**DOI:** 10.1038/srep11482

**Published:** 2015-07-14

**Authors:** Yang Mi, Yuxiang Weng

**Affiliations:** 1 Key Laboratory of Soft Matter Physics, Institute of Physics, Chinese Academy of Sciences (CAS), Beijing 100190, China

## Abstract

TiO_2_ is the most promising semiconductor for photocatalytic splitting of water for hydrogen and degradation of pollutants. The highly photocatalytic active form is its mixed phase of two polymorphs anatase and rutile rather than their pristine compositions. Such a synergetic effect is understood by the staggered band alignment favorable to spatial charge separation. However, electron migration in either direction between the two phases has been reported, the reason of which is still unknown. We determined the band alignment by a novel method, i.e., transient infrared absorption-excitation energy scanning spectra, showing their conduction bands being aligned, thus the electron migration direction is controlled by dynamical factors, such as varying the particle size of anatase, putting electron or hole scavengers on either the surface of anatase or rutile phases, or both. A quantitative criterion capable of predicting the migration direction under various conditions including particle size and surface chemical reactions is proposed, the predictions have been verified experimentally in several typical cases. This would give rise to a great potential in designing more effective titania photocatalysts.

TiO_2_ and TiO_2_-based materials have been prototypes for photocatalytic reactions^1^,^2^,^3^, since Fujishima and Honda discovered the photocatalytic splitting of water on a TiO_2_ electrode under ultraviolet light^4^. Despite enormous amount of research on these materials leading to many promising applications in solar energy conversion and photo-degradation of environmental pollutant related areas, continuous efforts have still been devoted to improve their photoactivities according to the basic principle of efficient charge separation, or controlled charge migration to prevent carrier recombination. Construction of heterojunction between composite semiconductors with staggered band alignment is often a successful strategy in controlling the photogenerated charge migration across the interface, where the electric potential provides the driving force for the controllable charge migration^5^.

An intriguing synergistic phenomenon for TiO_2_ is that the mixed-phase TiO_2_ of anatase and rutile exhibits higher photocatalytic activity than their pristine compositions. Although not fully understood, it is believed to involve charge migration which enhances the charge separation between the two phases. However, later experiments showed that the synergistic effect between anatase and rutile TiO_2_ that was observed in Degussa P25 was not universal, and the effect was related to the relative Fermi levels and shapes of anatase and rutile particles^6^, which indicates that the charge migration between the mixed phases is not unidirectional, depending on experimental conditions. A literature survey of the reported electron migration directions also concludes that the direction can be from rutile to anatase^7^,^8^,^9^,^10^ or anatase to rutile^11^,^12^,^13^,^14^,^15^. Apparently, the current models for the band alignment of rutile and anatase with a conduction band offset could hardly explain the bidirectional electron migration.

The bandgap of rutile and anatase TiO_2_ is of 3.0 and 3.2 eV respectively. Counting of all the possible alignments of their relative position of the conduction band (CB) and valence band (VB) levels, we notice that there exist five combinations as shown in Fig. 1. Explicitly, (1) staggered alignment with both of the CB and VB of anatase lying above those of rutile (type I)^15^,^16^; (2) staggered alignment with both of the CB and VB of rutile lying above those of anatase (type II)^17^,^18^; (3) included alignment (type III)^7^,^19^,^20^ (4) VBs are aligned (type IV)^21^,^22^; (5) CBs are aligned (type V)^23^,^24^. All the five combinations can find their corresponding proposed models in the literature with some of the models based on the experimental measurements or calculations of the corresponding relative energy levels. For example, Kavan *et al.* performed electrochemical measurements of flat band potentials for the rutile and anatase single crystals in solution separately, and concluded that the flat band of CB of anatase is 0.2 eV above that for rutile (type IV)^25^; Xiong *et al.* conducted the photoemission measurement of thin anatase film embedded with rutile nanocrystals, and found that the work function of the valence band of rutile is 0.2 eV lower than that of anatase, indicating that the conduction bands are aligned (Type V)^24^; Recently, Deák *et al.*^17^, and Scanlon *et al.*^18^, found theoretical indications for the type II staggered band alignment, and Scanlon *et al.* further performed X-ray photoemission spectroscopy (XPS) measurement of nanoparticulate structured rutile-anatase bilayer, supporting the calculated result.

The inconsistency for the experimentally determined band alignment of the mixed-phase TiO_2_ can mainly be attributed to the lack of the model TiO_2_ systems with well-defined anatase/rutile interfaces that are amenable to experimental techniques, since TiO_2_ thin film deposited stepwise onto a rutile substrate could hardly be forced into growing in anatase structure and vise versa^23^. To avoid such a sample problem, most recently, Pfeifer *et al.* employed a different strategy by growing rutile and anatase interfaces on a common semiconductor such as RuO_2_ and ITO^23^. In this way, the energy band alignment between anatase and rutile, as well as the band bending at the TiO_2_/RuO_2_ heterojunction can be derived from the XPS measurement. Although alignment of the band edges after band bending at the TiO_2_/RuO_2_ interface in their proposed model supports type II alignment, the flat band alignment free of band bending effect induced by TiO_2_/RuO_2_ heterojunction is apparently consistent to the type V alignment. Apparently, among all the experimental methods the key factor in accurate determination of the band alignment is to select a common reference energy level for both the anatase and rutile. Currently, the reference energy level could be the electropotential of the reference electrode in electrochemical measurements^25^, vacuum level in photoemission^24^ and core-level in XPS^18^,^23^.

In this work, we first determined the band alignment of anatase and rutile with our recently proposed method, i.e., transient infrared absorption-excitation energy scanning spectra (TIRA-ESS)^26^. The mid-gap transitions of interstitial defects Ti^3+^ to the localized excited states, which are proved to be the same within the experimental error (<0.02 eV) for both the anatase and rutile, are used as the reference energy levels for their band alignment. In this way, type V band alignment is derived with zero energy barrier for inter-phase electron migration. We then proposed a quantitative criterion based on dynamics for prediction of the electron migration directions under various conditions. Finally we confirmed the predicted controllable electron migration in a number of typical cases.

## Results and Discussion

### TIRA-ESS and mid-gap energy levels for rutile

**Figure 2:**
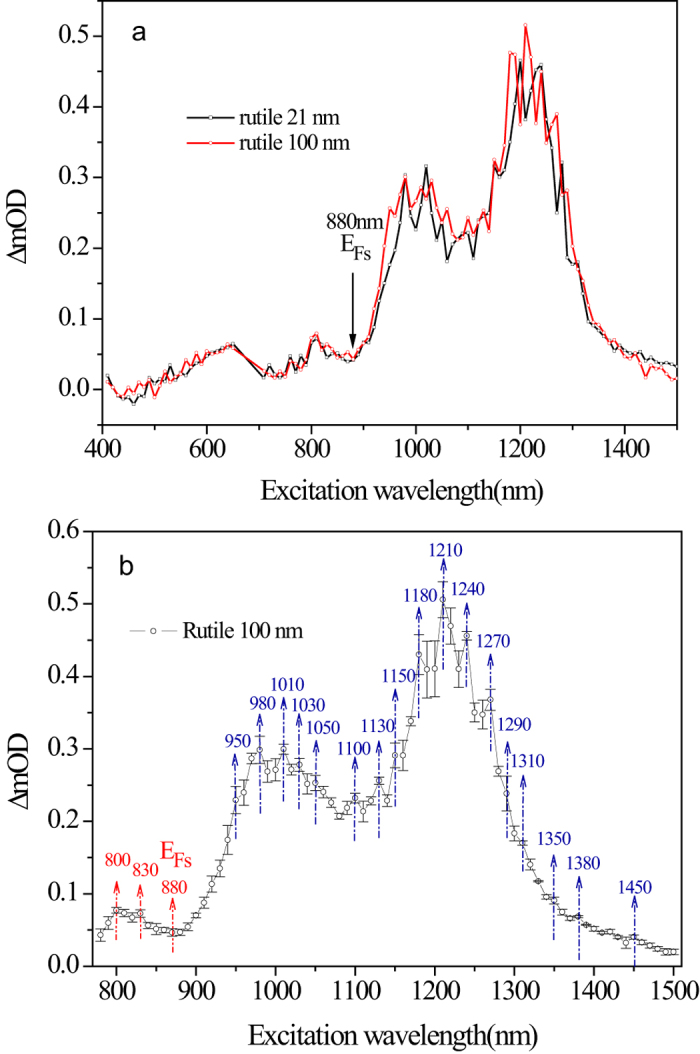
TIRA-ESS in vacuum for rutile nanoparticles. **a**, TIRA-ESS in the visible to NIR region for two different size of 21 and 100 nm in diameter. **b**, Averaged TIRA-ESS in vacuum of three independent measurements for 100-nm rutile nanoparticles in the NIR region. Standard errors were plotted as the error bars, the observed transition peak wavelengths assigned as the transitions from *E*_*Fs*_ (880 nm) to the localized excited states are labeled in blue, *E*_*Fs*_ and the other two levels of the trapped electrons below *E*_*Fs*_ are labeled in red. All the arrows are used as the guide for view.

Figure 2a shows the TIRA-ESS for 21-nm, 100-nm rutile nanocrystals respectively, together with Fermi level of the trapped electrons denoted as *E*_*Fs*_(*E*_*Fs*_is different from the Fermi level of the free electrons in CB denoted as *E*_*Fn*_^27^), we find that for the samples of two different size, the determined *E*_*Fs*_ are the same as 1.409 eV (880 nm) below the CB (Fig. S4-S5), and the TIRA-ESS are not affected by the particle size. Accordingly, those transitions with excitation energy larger than 1.409 eV (*λ* < 880 nm) having a slower decay kinetics are attributed to the deep trapped electrons being excited into the CB^26^,^28^, while the other transitions with energy smaller than 1.409 eV (*λ* > 880 nm) and a faster decay kinetics correspond to the excitation of the trapped electrons mainly at *E*_*Fs*_ to the localized excited states below the CB (Fig. S4). The discrete energy levels for the localized excited states can be determined from the observed NIR excitation peaks shown in Fig. 2b, which displays 17 peaks in NIR region. As we have pointed out previously that those transitions from the trapped electron below *E*_*Fs*_ might also contribute to the NIR transitions^26^, we noticed that below the *E*_*Fs*_ level, there are two trapped states, i.e., 830 and 800 nm, can be discerned. Thus we constructed a table to sort out the possible transitions from 830 and 800 nm levels which may overlap with those from the *E*_*Fs*_ level, and find only 3 among 17 observed transitions, i.e., at 1310, 1240 and 980 nm (see supplementary information and Table S1), which may not be the true localized excited energy levels.

### Assignment of the localized exited states

The attribution of the mid-gap energy levels has been investigated both experimentally and theoretically^29^,^30^. For the reduced TiO_2_, the surface trap states have been determined mainly as Ti^3+^ surface defects and oxygen vacancies. Besides, the Ti^3+^ also exists in the bulk as the interstitial defects^31^.

Ti^3+^ exhibits a broad absorption band in the visible to near-IR (NIR) region resulting from excitation of Ti^3+^-related bulk defects^32^. Khomenko *et al.* have examined the optical transitions and electron paramagnetic resonance (EPR) properties of reduced rutile single crystals^31^. In the NIR region, they detected two significant optical transitions peaked at 12000 cm^−1^ (833 nm) and 6500 cm^−1^ (1538 nm), and assigned these transitions to excitation of Ti^3+^ sites via small polaron type of charge transfer processes. Their results suggest that the main bandgap optical transitions in reduced TiO_2_ result from localized excitations of Ti^3+^ in bulk. Furthermore, photoluminescence in the NIR region has also been observed for both anatase and rutile. Fluorescence emission at 820 and 850 nm associated with Ti^3+^ interstitial ions in rutile single crystal have been reported by Gosh *et al.*^33^. Later Plugarus *et al.* investieged the cathod luminescence in NIR region for rutile and anatase polycrystal films, luminescence at 800 nm for anatase and 820 nm for rutile has been observed repectively, these emissions are related to Ti^3+^ ions in bulk^34^. Fernández *et al.* further investigated the cathod luminescence of defects in the deformed (110) and (100) surfaces of TiO_2_ single crystals, they concluded that the NIR emissions at 1.53 eV (810 nm) for both the crystals are from Ti^3+^ ions in bulk^35^. Santara *et al.*, reported photoluminescence emission from undoped TiO_2_ nanoribbons, which shows an emission peak at 1.47 eV (844 nm) independent of the TiO_2_ single phase or mixed phase, they suggest that Ti^4+^ interstitial defects are responsible for the NIR emission^36^. Therefore our observed transitions at 800 and 830 nm with energy larger than *E*_*Fs*_ could be arising from the transition of interstitial Ti^3+^ in bulk to the CB as envised by Komaguchi, *et al.*^13^, and the those NIR transitions with excitation energy less than *E*_*Fs*_ would be from the excitations of interstitial defect Ti^3+^ to the localized excited states below the CB.

**Figure 3:**
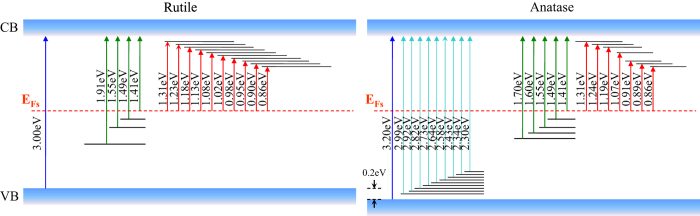
Experimentally determined mid-gap energy levels for anatase^22^ and rutile nanoparticles and the corresponding type V band alignment.

Compared TIRA-ESS in NIR region for both rutile and anatase^26^, there are 13 transitions being the same within the experimental error (10 nm) (Table S1), furthermore the observed *E*_*Fs*_ levels are also the same. Therefore, we conclude that the interstitial Ti^3+^ ions are almost the same for both the phases considering the energy resolution of our optical method (<0.02 eV), though EPR technique can tell the tiny difference of Ti^3+^ ions in the two different phases^19^. Thus, the relative energy levels for the transitions of interstitial Ti^3+^ to the localized excited states can be used as the inherent energy reference for the band alignment as shown in Fig. 3, which turns out to be a type V band alignment. When considering the band bending effect, it has been shown that for TiO_2_ with a diameter of 7.6 to 24 nm, the band bending from the center to the surface is only 0.004 V^37^, which is negligible for nanoparticles. We noticed that Hurum *et al.* has reported an activation energy for the electron transfer from rutile to anatase to be 8.3 × 10^−4^ eV^7^, which also supports our observed band alignment.

### Difference in surface states for anatase and rutile nanoparticles

**Figure 4:**
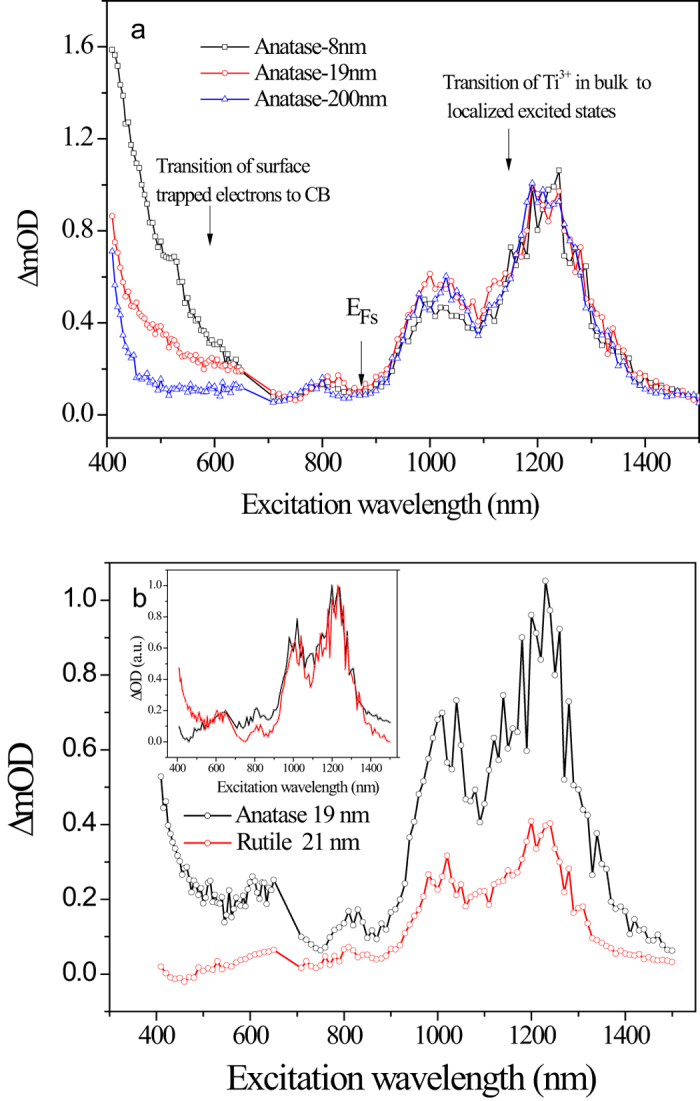
Comparison of TIRA-ESS in vacuum. **a**, TIRA-ESS for anatase nanoparticles of three different sizes of 8, 19 and 200 nm. **b**, TIRA-ESS for anatase and rutile nanoparticles of similar size around 20 nm, the inset shows the corresponding normalized spectra.

Figure 4a shows the TIRA-ESS for anatase nanocrystals of three different size, i.e., 8, 19 and 200 nm respectively, where the *E*_*Fs*_ is also not affected by the particle size, and the TIRA-ESS in the NIR region are almost independent of the particle size too. In contrast, those in the visible region arising from the transition from the deep trapped electrons to CB^26^ are significantly affected by the particle size in such a way, i.e., the smaller the particle size, the larger the density for the population of the deep trapped electrons. Furthermore their decay kinetics can be quenched effectively by oxygen^26^, therefore, the deep trapped electrons are directly related to the surface area of the particles, indicating that the deep trapped electrons mainly locate at the surface of the anatase nanoparticles.

Figure 4b compares the TIRA-ESS of anatase and rutile nanoparticles of similar size about 20 nm, where the normalized TIRA-ESS are shown in the inset. The normalized spectra reveal that the main difference lies in the visible range from 400 to 500 nm, where the rutile nanoparticles exhibits almost undetectable amount of deep surface trapped electrons with respect to the anatase nanoparticles, while very similar line-shapes for the interstitial Ti^3+^ transitions in NIR region are observed for both two polymorphs. Such a difference in the surface defects indicates that the anatase nanoparticles can provide large amount of surface oxygen vacancies for chemical adsorption while rutile nanoparticles could not.

### Criterion for predicting the direction of inter-phase electron migration

Based on the band alignment in Fig. 3, the electrons in the CB would cross the interface of the mixed-phase TiO_2_ in either direction without energy barrier, which indicates that the dynamical factors would come into play. Hurum *et al.* investigated charge migration using EPR in mixed-phase TiO_2_, they found that upon band-gap illumination, the photogenerated holes are exclusively trapped at the nanoparticle surface, while the photogenerated electrons are trapped within the bulk^8^. In line with this fact, Tamaki *et al.*, took the mobility of the photogenerated hole as zero^38^. Thus when considering the inter-phase charge flow, one can consider the electron flow only.

Considering a pulsed UV light such as 355 nm excitation of the mixed-phase TiO_2_, the extinction coefficients for both the anatase and rutile are almost the same^39^, therefore the initial photogenerated carrier concentration *c*_0_ can be set the same for both the phases. A significant difference is the carrier recombination rate in bulk, since rutile is a direct band-gap semiconductor while anatase is an indirect one, the carriers would recombine much faster for rutile than for anatase^40^. Thus we consider the following factors which would affect the inter-phase electron migration: (1) electron annihilation in bulk and (2) surface trapping of the free electrons, both processes compete with the electron migration; (3) difference in electron migration rate in the two phases.

Assuming that the sample is excited at *t*_*0*_ *=* 0 by a pulsed light, then observing the net out-flow of the photogenerated electrons *σ*(*t*) in a given phase after a period of time *t*. Further assuming that the electron migration is driven by a stochastic electric field built up by instant unbalanced charges at the interface, this stochastic electric field would revere its direction randomly in time, resulting in an equal mean intensity of the electric field 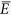 within the two phases and we have


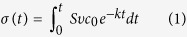


where *S* is the area of the interface, *k* is the electron annihilation rate and *υ* is the diffusion rate of the electrons in bulk, which can be expressed as 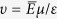, *μ* is the mobility of the electrons and *ε* is the dielectric constant^41^.

Taking an enough long observation time *t* (*t* = ∞), we have


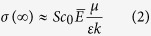


Obviously equation (2) shows that the direction of electron flow depends on relative magnitude of *μ*/*εk*, i.e., if (*μ*/*εk*)_*R*_ > (*μ*/*εk*)_*A*_ where the subscript “R” stands for “rutile” and “A” for “anatase” respectively, the photogenerated electrons would flow from rutile to anatase, and vise versa. Especially when (*μ*/*εk*)_*R*_ = (*μ*/*εk*)_*A*_, it indicates no net electron flow between the two phases, or the electrons would migrate in the two opposite directions with an equal probability. Therefore, the ratio between *μ*/*εk* for anatase and rutile becomes a quantitative criterion for judging the direction of the electron migration.

We noticed that the electron mobility for anatase at room temperature varies with its crystallinity^42^, from 17 cm^2^ V^−1^s^−1^ for large single crystals (a value measured in a single crystal cut at a typical size of 3 × 1 × 0.2 mm^3^, retrieved from Fig. 3 in reference 43)^42^,^43^ to 0.1 cm^2^ V^−1^s^−1^ for 20 nm nanocrystals^38^. For rutile, such a variation is much smaller, from a large single crystal (cut at 1 mm thick) of 0.5^44^ to 0.82 cm^2^ V^−1^s^−1^ (annealed at 80 °C) or 2.5 cm^2^ V^−1^s^−1^ (annealed at 450 °C)^45^ for 18-nm nanocrystals. The static dielectric constant for rutile and anatase is about 100 and 30 respectively^46^. Furthermore the electron annihilation rate depends on the surface states of the nanoparticles as well as surface chemical reactions, thus we discuss the direction of the electron flow under various conditions:

1. Large crystalline anatase and rutile in vacuum. Substituting *k*_*R*_ = 7.65 × 10^11^ *s*^−1^^32^; *μ*_*R*_ = 0.5 cm^2^ V^−1^ s^−1^ and *k*_*A*_ = 1.73 × 10^8 ^*s*^−1^^32^; *μ*_*A*_ = 17 cm^2^ V^−1^s^−1^ into equation (2), we find that (*μ*/*εk*)_*A*_ is about 5.1 × 10^5^ folds of (*μ*/*εk*)_*R*_, indicating that the electrons flow from the anatase to rutile.
2. Nanocrystalline rutile and anatase in vacuum. When varying the particle size, only the surface states and the mobility of anatase change significantly. For anatase nanoparticles with a diameter of 10–15 nm, the surface trapping rate for the photogenerated electrons has been reported to be 1/500 ps^−1^^38^, thus we take *k*_*A*_ = 2 × 10^9^*s*^−1^and *μ*_*A*_ = 0.1 cm^2^ V^−1^s^−1^, leading to (*μ*/*εk*)_*A*_of 17.3 (or 5.8) folds of (*μ*/*εk*)_*R*_, the value in parenthesis (here and the following) is calculated by taking *μ*_*R*_ = 2.5 cm^2^ V^−1^s^−1^, this suggests that the electron migration is still from anatase to rutile.
3. Nanocrystalline rutile and anatase with hole scavengers. In this case, the charge recombination is prevented for both anatase and rutile, and we assumes a same electron annihilation rate which leads to (*μ*/*εk*)_*A*_being 0.41 (or 0.14) fold of (*μ*/*εk*)_*R*_, suggesting a significant electron flow from rutile to anatase.
4. Nanocrystalline rutile and anatase with electron scavengers. When surface of the anatase is loaded with electron scavenger such as Pt, the electron transfer rate from the surface of TiO_2_ to the loaded Pt can be as fast as 1/2.3 ps^−1^^47^, thus we take a typical electron annihilation rate of 2 ps^−1^ when anatase surface is coupled to electron scavengers. We get (*μ*/*εk*)_*A*_ to be 0.61 (or 0.2) fold of (*μ*/*εk*)_*R*_, indicating an electron flow from rutile to anatase. All the parameters used and the corresponding results are summarized in Table S2 and S3.

For completeness, we calculated the ratio of (*μ*/*εk*)_*A*_ over (*μ*/*εk*)_*R*_ for all the four combinations of crystallinity (large single crystal and nanoparticles) for rutile and anatase under three different conditions, i.e., vacuum, with hole scavengers and with electron scavengers, the results are shown in Table S4. The predicted electron migration directions under 12 conditions are summarized in Table 1.

Based on the above predictions for the electron migration direction in the mixed-phase TiO_2_, it is not surprising that most of the reported cases with electrons flowing from anatase to rutile have been observed in vacuum^13^,^15^, while the reversed electron flow has been observed in photocatalytic systems coupled to either hole^7^ or electron^10^,^48^ scavengers, or to both^9^.

### Observation of controlled photogenerated electron migration in the mixed-phase TiO_2_

**Figure 5:**
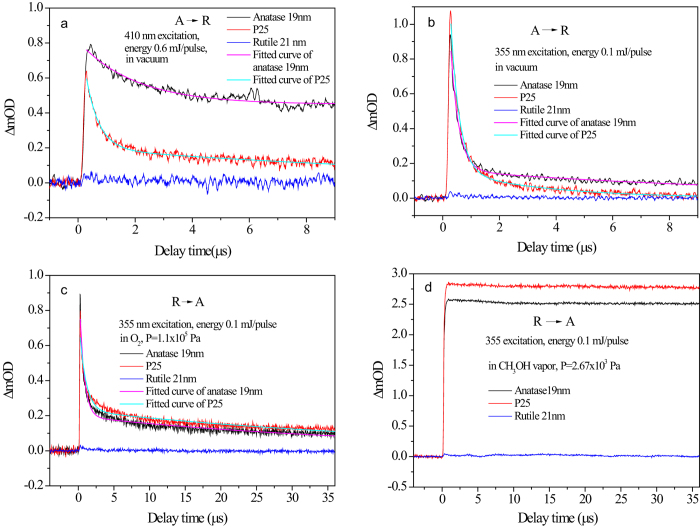
Experimental verification of the predicted electron migration directions in mixed-phase TiO_2_ by observing electron annihilation kinetics for three typical cases of vacuum, presence of electron scavenger, and hole scavenger. Transient mid IR decay kinetics for 19-nm anatase, 21-nm rutile and Degussa nanoparticle films in vacuum excited at (**a**) 410 nm (0.6 mJ/pulse) with fitting parameters for anatase *τ*_1_ = 2.0 *μs* (44.6%), *τ*_2_: non-decay (55.4%); P25 *τ*_1_ = 0.45 *μs* (80.0%), *τ*_2_ = 14.3 *μs* (20.0%); (**b**) 355 nm (0.1 mJ/pulse) with fitting parameters for anatase *τ*_1_ = 0.29 *μs* (92.1%), *τ*_2_ = 11.9 *μs* (7.9%); P25 *τ*_1_ = 0.31 *μs* (92.8%), *τ*_2_ = 3.4 *μs* (7.2%). **c**, 355 nm excitation (0.1 mJ/pulse) in 1 atm of O_2_ as electron scavenger with fitting parameters for anatase *τ*_1_ = 0.64 *μs* (81.5%)*τ*_2_ = 42.8 *μs* (18.5%); P25 *τ*_1_ = 0.89 *μs* (71.3%), *τ*_2_ = 49.0 *μs* (28.7%)., **d**, 355 nm (0.1 mJ/pulse) in 20 torr of CH_3_OH vapor as hole scavenger. Where the percentages in the parentheses are the relative contributions of the corresponding exponential decay components.

Figure 5a,b show the photogenerated free electron decay kinetics probed by mid IR for 19-nm anatase, 21-nm rutile and Degussa nanoparticle films in vacuum excited at 410 and 355 nm respectively. The results reveal that almost no detectable electrons in rutile is observed owing to the fast recombination of the photogenerated carriers in ps time domain^32^, while the photogenerated carriers in Degussa recombine much faster (410 nm excitation: *τ*_1_ = 0.45 *μs*, *τ*_2_ = 14.3 *μs*; 355 nm excitation: *τ*_1_ = 0.31 *μs*, *τ*_2_ = 3.4 *μs*) than that in pure anatase (410 nm excitation: *τ*_1_ = 2.0 *μs*, *τ*_2_: non-decay; 355 nm excitation *τ*_1_ = 0.29 *μs*, *τ*_2_ = 11.9 *μs*), indicating that the photogenerated electrons in anatase phase has been substantial quenched by rutile, giving a clear evidence that the photogenerated electrons migrate from anatase to rutile.

Figure 5c,d compare the electron decay kinetics excited at 355 nm for 19-nm anatase, 21-nm rutile and Degussa nanoparticle films in the presence of O_2_ as the electron scavenger^49^ and methanol as the hole scavenger^7^ respectively. For the former, a slower recombination process in Degussa (*τ*_1_ = 0.89 *μs*, *τ*_2_ = 49.0 *μs*) with respect to that in pure anatase (*τ*_1_ = 0.64 *μs*, *τ*_2_ = 42.8 *μs*) has been observed, while for the latter a significant increase in the amplitude has been observed. They both indicate that the electrons migrate from rutile to anatase. It should be noted that the capture reaction of photoinduced holes by methanol complete within 1 ns, and lifetime of photogenerated electrons can be prolonged to seconds^50^. However, the mid IR kinetics for rutile in Fig. 5d shows a negligible IR absorbance. Our observation is similar to that reported in a recent work, where the authors gave a possible account for the absence of mid IR absorbance due to the extremely low extinction coefficient of electrons in rutile^15^.

In conclusion, we have determined the mid-gap optical transitions arising from the Ti^3+^ interstitial defects in bulk for rutile, which are same as those for antase. The band alignment for rutile and anatase is derived showing that CBs are aligned. Using these mid-gap energy levels as the inherent energy references, a quantitative criterion based on dynamics for prediction of electron migration in the mixed-phase TiO_2_ under various conditions has been proposed, which opens a great space to control the direction of the electron migration. Significant difference in deep surface trapped states has been demonstrated for rutile and anatase, which would also account for the different photocatalytic activity for these two polymorphs.

## Methods

Anatase, rutile TiO_2_ and Degussa P25 nanoparticles were commercially available and examined by XRD for the purity and mean size. The mean sizes of TiO_2_ nanoparticles were calculated by Sherrer’s equation (Supporting information, Fig. S1-S3). The nanocrystal films for TIRA-ESS measurement were prepared following the reported procedure^51^. Details of the set-up for TIRA-ESS has been described elsewhere^26^. Briefly, a quantum cascade laser continuously tunable from 4.69 to 4.88 μm, (TLC-21045, Daylight Solutions) was used as the mid-IR probe light, while 355 nm laser pulses from a Nd:YAG laser (Quanta Ray, Spectra Physics) with a pulse duration of 10 ns and a repetition rate of 10 Hz were used to pump an optical parametric oscillator (GWU premiScan-ULD/240, Spectra Physics) which acted as a wavelength-scanning excitation source (output signal beam tunable from 410 to 709 nm, and idler beam from 710 to 2630 nm) to excite the mid-gap states.

The principle of TIRA-ESS is to scan the excitation energy within the band gap of TiO_2_ from the visible to near IR region, and use the transient mid-IR-difference spectra to probe the photo-excited electrons within the conduction band or at the localized excited states below the CB. Both the CB electrons and the excited localized electrons can be detected by the mid-IR probe and distinguished by their remarkable difference in the corresponding absorption spectrum and the decay kinetics (slower decay for the former and faster decay for the latter)^26^. All the TIRA-ESS, if not specified, were probed at 2090 cm^−1^ at a delay time of 250 ns after the excitation pulse in a chamber with a vacuum of 1.0 × 10^−6^ mbar. The excitation energy is 0.6 mJ/pulse with a beam size of 4 mm in diameter, and the IR absorbance has been scaled by the excitation intensity in terms of the number of photons (10^12^ per pulse). All the kinetics were also probed at 2090 cm^−1^.

## Additional Information

**How to cite this article**: Mi, Y. and Weng, Y. Band Alignment and Controllable Electron Migration between Rutile and Anatase TiO_2_. *Sci. Rep.* **5**, 11482; doi: 10.1038/srep11482 (2015).

## Supplementary Material

Supplementary Information

## Figures and Tables

**Figure 1:**
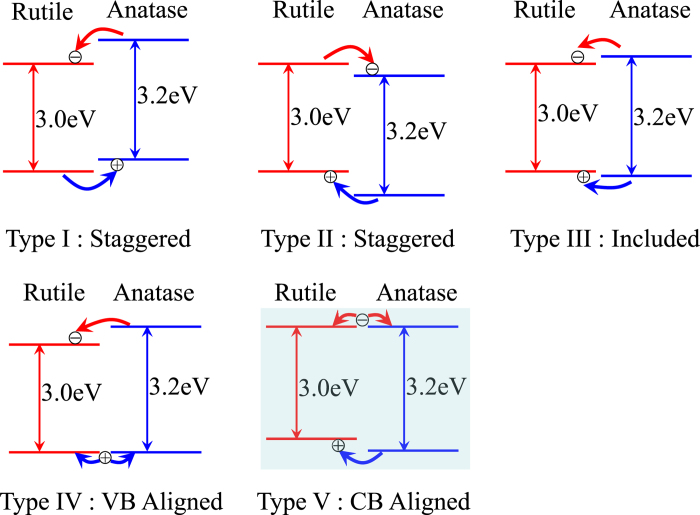
Schematic diagrams showing five possible band alignments between rutile and anatase. Our result supports the highlighted type V alignment.

**Table 1:** Predicted directions for electron migration in the mixed-phase TiO_2_ under various conditions.

Direction of electron flow			
Morphology	Vacuum	h-scavenger	e-scavenger
A_crystal_/R_crystal_	A→R	A→R	A→R
A_nano_/R_crystal_	A→R	R→A	A⇋R
A_crystal_/R_nano_	A→R	A→R	A→R
A_nano_/R_nano_	A→R	R→A	R→A

“A” stands for anatase, “R” for rutile; “h” for hole and “e” for electron; “⇋” indicates that electrons migrate in both directions with an equal probability.
