# Supplementary material for: Band Alignment and Controllable Electron Migration between Rutile and Anatase TiO2
Source: Sci Rep. 2015 Jul 14;5:11482. doi: 10.1038/srep11482 (PMC4500998; doi:10.1038/srep11482)
Supplement: Supplementary Information [file srep11482-s1.pdf]

Supplementary information

Band Alignment and Controllable Electron Migration between Rutile and Anatase TiO_2

Yang Mi, Yuxiang Weng*

Key Laboratory of Soft Matter Physics, Institute of Physics, Chinese Academy of Sciences (CAS), Beijing 100190, China.

Mail to: yxweng@aphy.iphy.ac.cn

Table of contents

X-ray diffraction analysis of the TiO_2 powders ……………………………………1
Figure S1 …………………………………………………………………………...1
Figure S2 …………………………………………………………………………...2
Figure S3 …………………………………………………………………………...2
Determination of Fermi level of the trapped electrons for rutile ………………......3
Figure S4 …………………………………………………………………………...4
Figure S4 …………………………………………………………………………...5
Table for searching the possible transitions from the energy levels below E_{Fs} contributing to the observed NIR peaks in TIRA-ESS.......…………………………..6
Table S1……………………………………………………………………………7
Calculation of ratios of $\mu / \varepsilon k$ for anatase over rutile………………………………..8
Table S2……………………………………………………………………………..9
Table S3……………………………………………………………………………..9
Table S4…………………… ………………………………………………………...10

X-ray diffraction analysis of the TiO_2 powders

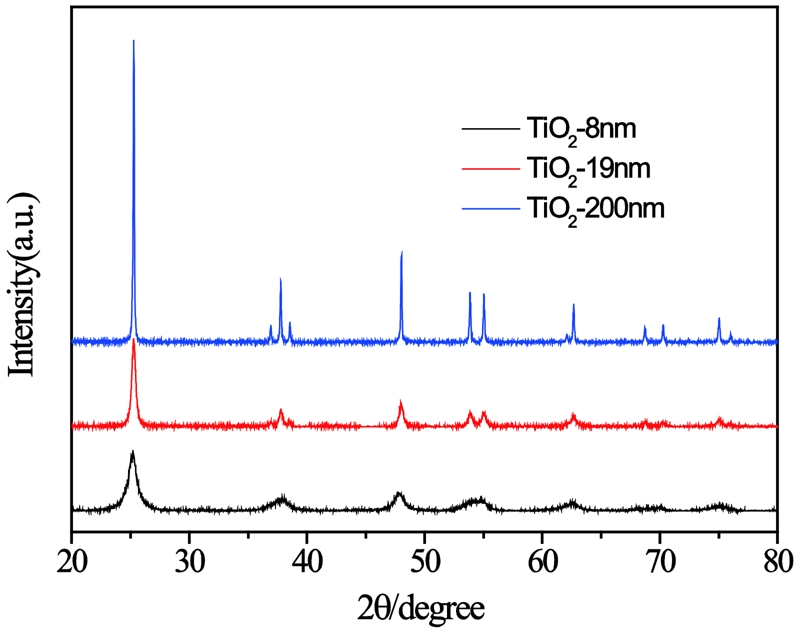

Figure S1 | X-ray diffraction patterns for anatase TiO_2 powders of three different sizes. X-ray diffraction patterns were recorded on a diffractometer (Rigaku D/max-2500, Japan) using CuKa radiation at 45kV and 250 mA and a scan rate of 0.1° 2θ per second. All peaks are in good agreement with the standard spectrum of anatase TiO_2 (JCPDS 21-1272), indicating TiO_2 in the anatase phase. The nanocrystal size was calculated to be 8 and 19 nm by Scherrer equation $D = K\lambda/B \cos \theta$, the 200-nm TiO_2 powder was indicated as received.

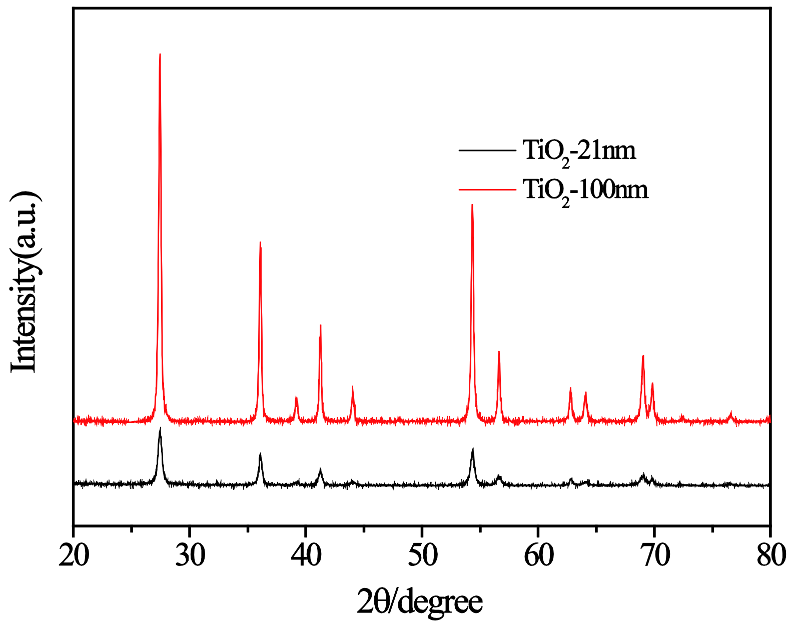

Figure S2│ X-ray diffraction patterns of rutile TiO_2 powders of two different sizes. All peaks are in good agreement with the standard spectrum of rutile TiO_2 (JCPDS 21-1276), indicating TiO_2 in the rutile phase. The nanocrystal sizes were calculated to be 21 nm and 100 nm by Scherrer equation $D = K\lambda/B \cos \theta$.

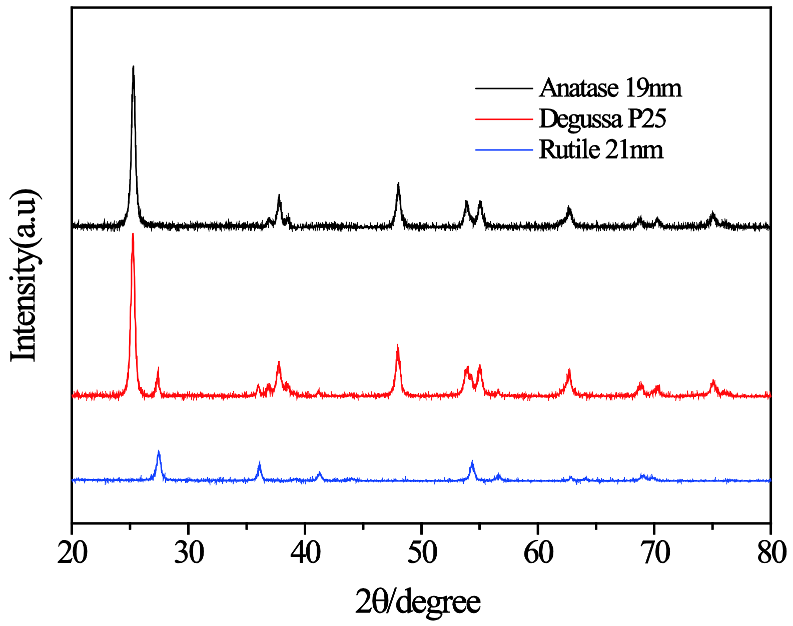

Figure S3│ X-ray diffraction patterns of mixed-phase TiO_2 Degussa P25 Powder in comparison with those of anatase and rutile phases having a similar particle size.

Determination of Fermi level of the trapped electrons for rutile

The Fermi level of the trapped electrons E_{Fs} is defined as the matching point at which the minimum energy needed for excitation of the trapped electrons to the bottom of the CB being equal to the maximum energy needed for excitation of the trapped electrons to the top of the localized excited energy levels immediately below the bottom of the CB. The electrons in the conduction band would have a slower decay kinetics (355 nm excitation in Fig. S4), whereas the electrons in the localized excited states would have a faster decay kinetics (1240 nm excitation in Fig. S4), and the excitation energy for the E_{Fs} would correspond to a transition point at which the slower decay kinetics begin to change to the faster decay kinetics as shown in Fig. S5, which shows that when excited at 880 nm, the kinetics mainly consists of slower decay component, the faster decay component becomes obvious when the excitation wavelength is scanned to 890 nm. The biphasic feature at 890 nm indicates that the trapped electrons at localized excited states are in thermal equilibrium with the CB electrons. Therefore the E_{Fs} would be at 880 nm excitation, or 1.409 eV below the bottom of the CB.

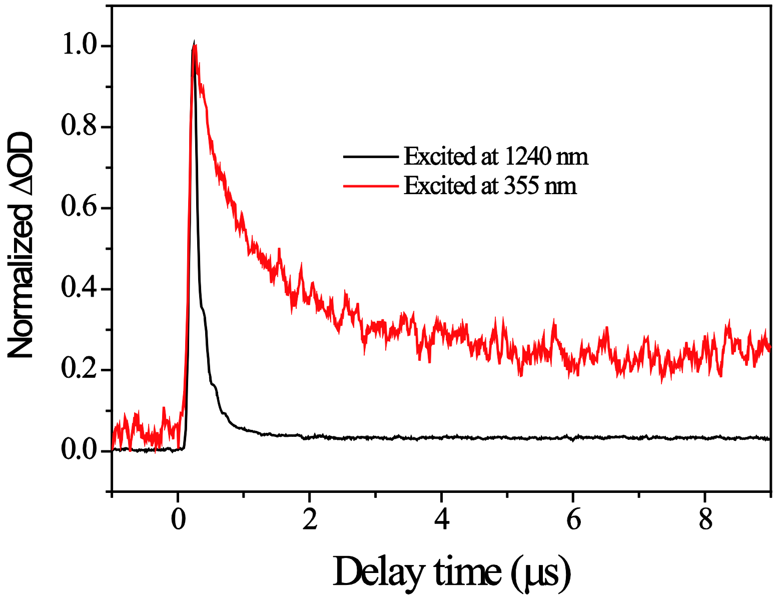

Figure S4 | **Comparison of two types of typical normalized decay kinetics in vacuum for free carriers in conduction band excited at 355 nm and localized excited states excited at 1240 nm**. Excitation energy: 0.6 mJ/pulse.

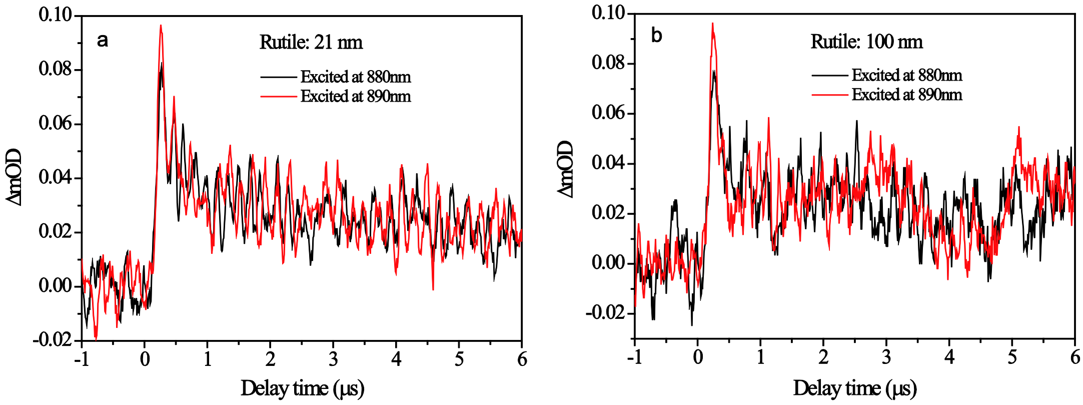

Figure S5 | Search of E_{Fs} by finding a transition in kinetics from the free electron decay to the decay of the localized excited states by changing the excitation energy. Two typical decay kinetics excited at 880 and 890 nm probed in vacuum for 21 nm (**a**) and 100 nm (**b**) rutile nanoparticles respectively. Excitation energy: 0.1 mJ/pulse.

Table for searching the possible transitions from the energy levels below E_{Fs} contributing to the observed NIR peaks in TIRA-ESS

The experimentally observed NIR transitions of Ti^{3+} in bulk to the localized excited states for rutile are listed in first column in Table S1， while the second column shows the calculated uncertainty in eV at the given wavelength (5 nm, since the lower energy state of transition is fixed). The third column lists the transition energy assuming all the transitions starting at E_{Fs}. The fourth and fifth columns list the calculated transition energies started at 1.494eV (830 nm) and 1.550 eV (800 nm) below E_{Fs} obtained by adding the corresponding ΔE in the first raw to the fourth and fifth columns respectively. If the transition energies expected from these two starting levels coincide with those observed transition energy in the third column within the uncertainty at the given wavelengths, then the observed transitions could be from those below the E_{Fs}. Therefore the observed transition at 1240 nm could be from 1.494 eV (830 nm) to an already existing level of 1350 nm, or from 1.550 eV (800 nm) to an already existing level of 1450 nm, all these transitions have the same energy around 1.000 eV marked as red in the table S1. Therefore the observed transition at 1240 nm together with other two additional transitions at 1130 nm (marked in green) and 980 nm (marked in blue) may not be the true energy levels of the localized excited states. The last column lists all the observed transitions of the trapped electrons to the localized excited states in anatase crystalline film reported previously in reference 26, apparently they match well with those observed in rutile within the experimental error (10 nm).

Table S1. Observed Transition Wavelengths Correlated to the Transition Energies with Starting Energy Levels at E_{Fs} and Two Other Trapped Electron Energy Levels below E_{Fs}

	$\Delta E = E_{Fs} - E_{trap}$	0.000eV	0.085eV	0.141eV	
Trap state / Rutile Observed transitions	Uncertainty in eV For 5nm in wavelength	1.409 eV E_{Fs} (880nm)	1.494eV (830 nm)	1.550 eV (800 nm)	Anatase observed transitions
1450nm	0.00294	0.85517	0.94017	0.99617	1450 (nm)
1380nm	0.00324	0.89855	0.98355	1.03955	1390 (nm)
1350nm	0.00339	0.91852	1.00352	1.05952	1360 (nm)
1310nm	0.0036	0.94656	1.03156	1.08756	1310 (nm)
1290nm	0.00371	0.96124	1.04624	1.10224	
1270nm	0.00383	0.97638	1.06138	1.11738	1270 (nm)
1240nm	0.00402	1.00000	1.08500	1.14100	1240 (nm)
1210nm	0.00422	1.02479	1.10979	1.16579	
1180nm	0.00443	1.05085	1.13585	1.19185	
1150nm	0.00467	1.07826	1.16326	1.21926	1160 (nm)
1130nm	0.00483	1.09735	1.18235	1.23835	1130 (nm)
1100nm	0.0051	1.12727	1.21227	1.26827	1080 (nm)
1050nm	0.0056	1.18095	1.26595	1.32195	
1030nm	0.00582	1.20388	1.28888	1.34488	1040 (nm)
1010nm	0.00605	1.22772	1.31272	1.36872	1000 (nm)
980nm	0.00642	1.26530	1.35030	1.40630	970 (nm)
950nm	0.00683	1.30526	1.39026	1.44626	950 (nm)

The coincidences of the transition energies are grouped with a specific color.

Calculation of ratios of $\mu / \varepsilon k$ for anatase over rutile

All the values in parenthesis in Table S3 and S4 refer to the calculated results of using the mobility of rutile nanocrystal having a value of 2.5 in the parenthesis of Table S1.

Table S2. Physical parameters and electron annihilation rates under various conditions for rutile and anatase

**Parameters**	**Condition**	**Rutile**	**Anatase**
Dielectric constant ε	large crystal/ nanocrystal	100	30
Mobility μ ($cm^2 V^{-1}s^{-1}$)	large crystal	0.5	17
	nanocrystal	0.82 (2.5)	0.1
Electron annihilation rate k	large crystal (vacuum)	$7.65\times10^{11} s^{-1}$	$1.73\times10^{8} s^{-1}$
	nanocrystal (vacuum)	$7.65\times10^{11} s^{-1}$	$2\times10^{9} s^{-1}$
	large crystal (h- scavenger)	k_{hs}	k_{hs}
	Nanocrystal (h- scavenger)	k_{hs}	k_{hs}
	large crystal (e- scavenger)	$7.65\times10^{11} s^{-1}$	$5\times10^{11} s^{-1}$
	Nanocrystal (e-scavenger)	$7.65\times10^{11} s^{-1}$	$5\times10^{11} s^{-1}$

Table S3 Calculated values of $\mu/\varepsilon k$ for rutile and anatase under various conditions

**Condition**	$\frac{\mu}{\varepsilon k}$ (rutile)	$\frac{\mu}{\varepsilon k}$ (anatase)
large crystal vacuum	6.5×10^{-15}	3.3×10^{-9}
Nanocrystal vacuum	1.1×10^{-14} (3.3×10^{-14})	1.7×10^{-12}
h-scavenger large crystal	$5.0\times10^{-3}/k_{hs}$	$0.57/ k_{hs}$
h-scavenger nanocrystal	$8.2\times10^{-3}/k_{hs}$ ($2.5\times10^{-2}/k_{hs}$)	$3.3\times10^{-3}/k_{hs}$
e-scavenger large crystal	6.5×10^{-15}	1.1×10^{-12}
e-scavenger nanocrystal	1.1×10^{-14} (3.3×10^{-14})	6.7×10^{-15}

Table S4. Calculated ratios of $\mu/\varepsilon k$ for anatase over rutile and under various conditions

**Mixed-phase morphology**	**ratio of** $(\mu/\varepsilon k)_A/(\mu/\varepsilon k)_R$		
	Vacuum	h-scavenger	e-scavenger
$A_{crystal}/R_{crystal}$	5.1×10^{5}	1.1×10^{2}	169
$A_{nano}/R_{crystal}$	2.6×10^{2}	0.66	1.0
$A_{crystal}/R_{nano}$	3.0×10^{5} (1.0×10^{5})	70 (23)	100 (33)
A_{nano}/R_{nano}	154(51)	0.41 (0.14)	0.61 (0.2)
